# The Role of Polymorphonuclear Leukocyte Counts from Urethra, Cervix, and Vaginal Wet Mount in Diagnosis of Nongonococcal Lower Genital Tract Infection

**DOI:** 10.1155/2018/8236575

**Published:** 2018-07-26

**Authors:** Ivana Randjelovic, Amir Moghaddam, Birgitte Freiesleben de Blasio, Harald Moi

**Affiliations:** ^1^Oslo University Hospital, Department of Venereology, The Olafia Clinic, Norwegian National Advisory Unit on Sexually Transmitted Infections, Oslo, Norway; ^2^Fürst Medisinsk Laboratorium, Oslo, Norway; ^3^Department of Infectious Disease Epidemiology and Modelling, Norwegian Institute of Public Health, Oslo, Norway; ^4^Department of Biostatistics, Oslo Centre for Biostatistics and Epidemiology, Institute of Basic Medical Sciences, University of Oslo, Oslo, Norway; ^5^Faculty of Medicine, University of Oslo, Oslo, Norway

## Abstract

**Objective:**

The aim of this study was to evaluate whether the polymorphonuclear leukocyte (PMNL) inflammatory response in women with nongonococcal lower genital tract infection (LGTI) can be used to optimize criteria for syndromic treatment.

**Methods:**

A cross-sectional study of 375 women attending the STI clinic in Oslo. Urethral, cervical, and vaginal specimens underwent microscopy for PMNLs. Chlamydia trachomatis (Ct) and other STIs were detected in the cervical/vaginal swabs and urine, using nucleic acid amplification test (NAAT). After excluding vulvovaginal candidiasis, genital herpes, and trichomoniasis, we correlated clinical and microscopic signs of inflammation with positive NAAT for Ct, mycoplasma genitalium (Mg), and Ureaplasma urealyticum (Uu) in a subgroup of 293 women.

**Results:**

To predict a positive Ct, the combination of high cut-off urethritis (≥10 PMNLs/HPF) and microscopic cervicitis had a high specificity of 0.93, a PPV of 0.37, and a sensitivity of 0.35. LGTI criteria had low predicting values for Mg and Uu.

**Conclusion:**

Including microscopic criteria for the diagnosis of LGTI gives better indication for presumptive antibiotic treatment than anamnestic and clinical diagnosis alone.

## 1. Introduction

Lower genital tract infection (LGTI) is a broad definition that includes clinical and microscopic cervicitis, purulent vaginal wet mount, and urethritis. Cervicitis was first recognized as an important clinical entity in 1984 by Brunham et al. and was described as the female counterpart to urethritis in men [1]. More than twenty years later cervicitis is no longer ignored but still misunderstood, mainly because of controversial clinical and microscopic diagnostic criteria [2].Two major diagnostic signs characterize clinical cervicitis: (1) a purulent or mucopurulent endocervical exudate visible in the endocervical canal or by an endocervical swab specimen and (2) sustained endocervical bleeding easily induced by gentle passage of a cotton swab through the cervical os. One or both signs may be present [3]. Sexually transmissible pathogens must always be suspected as a cause of cervicitis; however nonspecific cervicitis is also common. The CDC Sexually Transmitted Diseases Treatment Guidelines recommend presumptive treatment for clinical cervicitis with antimicrobials against Chlamydia trachomatis (Ct) and Neisseria gonorrhoeae (Ng) for women at increased risk, especially if followup cannot be ensured, or if testing with NAAT is not possible [3]. The cervical immune response includes infiltration by polymorphonuclear leukocytes (PMNLs) which can be semiquantified. Consequently, an increased number of PMNLs of more than 30 per 1000x high power field (>30 PMNLs/HPF) in a stained cervical smear in combination with at least one clinical criterion has been used as a definition of cervicitis [4, 5]. Unlike cervicitis, female urethritis is seldom looked for, despite its connection with Ct infection [3, 4, 6]. In the absence of the major diagnostic signs of inflammatory vaginitis, purulent wet mount might be a sensitive indicator of cervical inflammation with a high negative predictive value (i.e., cervicitis is an unlikely condition if normal wet mount) [3, 5]. Purulent wet mount has been associated with both Ct and Ng infection of the cervix [3].

The lack of good criteria for the diagnosis of LGTI is a clinical dilemma, because syndromic treatment results in overuse of antibiotics, which prompts the development of antibiotic-resistant bacteria. The aim of this study was to evaluate factors for inflammatory response in LGTI and assess which combination of clinical and microscopic criteria for LGTI gives the highest sensitivity and positive predictive value (PPV) in the diagnosis of Ct to optimize the criteria for treatment nonspecific LGTI.

## 2. Methods

### 2.1. Study Population

Between April 2011 and March 2012 a convenience sample of female patients attending a “drop in” venereology clinic (the Olafia Clinic) in Oslo was assessed. Eligibility criteria included having had one or more male sexual partners in the preceding six months, not being pregnant, and having no menstrual bleeding at the time of examination. Four hundred and four women were considered eligible for enrollment and gave written informed consent in this cross-sectional study. Twenty-nine were excluded because of insufficient tests, positive test for Ng, or missing microscopy results, leaving 375 women eligible for the study. Self-reported behavioural variables for the last 6 months (condom use for vaginal and anal sex, number of sexual partners, regular and new partners, current smoking, current use of combined oral contraceptive (COC), and current use of injectable depot medroxyprogesterone acetate (DMPA)) and current genital tract symptoms (lower abdominal pain, dysuria, itching/burning, vaginal discharge, rash, blisters/ ulcers, and warts) were collected in accordance with the study protocol.

For the purpose of making the analysis as clinically relevant as possible, we excluded seven women with clinical genital herpes, sixty-seven microscopic verified vulvovaginal yeast infections, one trichomoniasis, and two women with dual infection (yeast and herpes). Five women lacking vaginal wet-mount microscopy results were also excluded. Thus 293 women were included in the evaluation of clinical and microscopic criteria for LGTI. 

### 2.2. Sampling and Laboratory Methods

The cervix was examined for clinical signs of inflammation: presence of a yellow discharge or easily induced cervical bleeding (friability). If one or both of these signs were present, a clinical diagnosis of cervicitis was registered.

After cleaning the cervix with a cotton swab, a specimen for the detection of Ct,* Mycoplasma genitalium *(Mg),* Mycoplasma hominis* (Mh),* Ureaplasma urealyticum* (Uu),* Ureaplasma parvum* (Up), and* Trichomonas vaginalis* (Tv) was taken with the same swab from the cervical canal, the portio surface, vaginal wall, and vestibulum. In addition, 10-20 ml of first void urine was collected. DNA was isolated from 200 *μ*l of medium from the cervical swab (Roche Molecular system) and from 200 *μ*l of first void urine (FVU) [7]. NAAT was performed for each of these microorganisms from FVU, as well as from a cervical/vaginal swab. If positive in one specimen the test was regarded as a true positive. In cases of clinically suspected genital herpes, a NAAT for HSV was taken.

Urethral smears were taken with a sterile blunt curette and cervical specimens for microscopic smears were taken with a cotton swab. Both were flame-fixated, stained with methylene blue, and examined with a Nikon Eclipse E400 microscope 1000 x oil emersion (HPF). For the microscopic definition of cervicitis we used >30 PMNLs/HPF observed in at least five visual fields with the highest concentration in cervical smears. There is no established microscopic definition of female urethritis. We used the same cut-off as recommended in males: low cut-off urethritis ≥5 PMNLs/HPF [8] as well as a high cut-off urethritis ≥10 PMNLs/HPF [9].

For wet mount examination, samples of vaginal discharge were collected from the vaginal wall with a 10 *μ*l plastic loop and diluted in 20% KOH and 0.9% NaCl, respectively. These vaginal wet mounts were examined with a phase-contrast microscope (× 400). The number of white blood cells (WBCs) in relation to the number of epithelial cells (WBS < = > epithelial cells) was recorded, in addition to signs of bacterial vaginosis (BV) [10]. Yeast infection was diagnosed microscopically with KOH wet mount. Wet mount examinations were carried out by the examining during the patient's visit and recorded before the result of the NAATs was available. WBCs>epithelial cells was diagnosed as a purulent wet mount.

Vaginal specimens were also tested for pH with a paper strip (Macherey-Nagel pH paper 3.8-5.8) and a whiff-amine test after adding 20% KOH to a drop of vaginal discharge.

### 2.3. Statistical Analyses

Association of all variables with cervicitis was estimated using log binomial regression and results are presented in terms of prevalence ratios (PRs) with p values based on *χ*2 testing. Adjusted PRs were estimated by multivariate log binomial regression and forward selection of significant variables (p<0.05). Prior to the multivariate analyses, missing data in the relevant variables (range: 0-10%) were imputed using logistic regression in order to retain available information in the data and allow for model comparison. Sensitivity, specificity, PPV, and NPV are presented with 95% CI based on exact binomial limits. Number needed to diagnose (NND) is defined as the number of patients that must be tested, in order to produce one correctly classified positive test; the NND may be negative in situations where the diagnostic test performs poorly. All data were analyzed using the epiR, mice and logbin packages under version 3.2.5 of R (The R Foundation for Statistical Computing, Vienna, Austria). The stained slides were blinded and confirmed by an independent observer (IR). Cohen's *κ* was calculated to determine the agreement between the two evaluations and revealed a moderate to good agreement. For cervicitis >30 PMNLs/HPF: *κ* = 0.542 (95% CI 0.450-0.632) p<0.001 and for urethritis ≥5 PMNLs/HPF: *κ* = 0.639 (95% CI 0.561-0.717) p<0.001.

## 3. Results

A total of 375 women with a median age of 25 years (range: 17-53 years) were included. Half of them (190) reported vaginal discharge and 75 (20%) dysuria. Clinical signs of cervicitis were detected in 100 (27%) women and microscopic cervicitis in 122 (33%). Low cut-off urethritis was present in 176 (47%), high cut-off urethritis in 69 (18%), and purulent wet mount in 85/370 (23%).

The prevalence of a positive NAAT result was as follows: Ct 39/375 (10%), Mg 16/372 (4%), Mh 90/375 (24%), Uu 91/375 (24%), Up 299/375 (80%), and Tv 1/375 (0.3%) (Table 1). There were 20 dual infections with Ct/Mg/Uu, of which 11 (55%), 8 (40%), and 1 (5%) involved Ct/Uu, Uu/Mg and Ct/Mg, respectively. Bacterial vaginosis (BV) was diagnosed in 110 women (29%), and yeast was detected with microscopy in 69 (18%) women. Nine women were positive for HSV using NAAT.

### 3.1. Univariate and Adjusted Prevalence Ratio of all Included Women

We assessed the association between microscopic signs of LGTI and sociodemographic/biological characteristics including sexual behaviour and found a statistical significant increased prevalence ratio (PR) of microscopic cervicitis in women who had a regular or cohabiting partner in the last 6 months (Table 1). This was confirmed in the adjusted analysis (Table 2). A decreased risk of purulent wet mount with condom use (Table 1) was not confirmed in the adjusted analysis (Table 2). Patients with regular partners used condoms less often than patients without regular partners (21% vs 40%, p=0.0015). There was no association of microscopic signs of LGTI with age, age of sexual debut, anal sex, contraceptive use, BV, and previous STIs including Ct and Mg or smoking. The reported symptoms such as lower abdominal pain, dyspareunia, genital rash, itching, or vesicular lesions/ulceration were not associated with microscopic LGTI parameters. There was a highly significant association between Ct and all microscopic signs of LGTI (Table 1), which was confirmed in the adjusted analyses (Table 2). Such association was not found for Mg and Up, or dual infections, in the adjusted analyses (Table 2). Vulvovaginal candidiasis was significantly correlated with high grade urethritis (p=0.009) and with purulent wet mount (p=0.001) (Table 1). Uu infection was significant associated with microscopic cervicitis in the univariate analysis, but not in the adjusted PR. There was no association between previous STIs and microscopic parameters (Table 1).

In univariate analysis, there was highly significant association between all clinical signs of cervicitis and purulent wet mount (p<0.001), as well as between mucopurulent endocervical exudate and both degrees of urethritis (p<0.041 and p<0.010, respectively), and a negative association between purulent wet mount and no clinical signs of cervicitis (Table 1).

There was a statistical significant adjusted association between dysuria and high cut-off urethritis (PR=1.66 (1.17-2.36), (p=0.004)) (Table 2) and between reported symptoms of vaginal discharge and purulent wet mount (PR=1.64 (1.15-2.33), (p=0.006)).

### 3.2. Evaluation of Clinical and Microscopic Criteria for LGTI in the Subgroup

A subgroup of 293 women was selected for the evaluation of the best criteria for presumptive treatment with antimicrobials effective against Ct (see study population). The median age (25 years) in the subgroup was the same as in the total group. Prevalence of positive NAATs for STIs in the subgroup was similar as in the total group: Ct 31 (11%), Mg 13 (4%), and Uu 67 (23%).

We correlated positive NAATs for Ct, Mg, and Uu with all signs of LGTI (Table 3). For predicting a positive Ct, low and high cut-off urethritis had a sensitivity = 0.77 (0.59-0.9) and 0.42 (0.25-0.61), specificity = 0.58 (0.51-0.64) and 0.86 (0.81-0.90), and positive predictive value (PPV) = 0.18 and 0.26, respectively.

For predicting a positive Ct, clinical and microscopic cervicitis had a sensitivity = 0.42 (0.25-0.61) and 0.52 (0.33-0.70), specificity = 0.78 (0.72-0.83) and 0.71 (0.65-0.77), and PPV = 0.18 and 0.18, respectively. Purulent vaginal wet mount had a sensitivity = 0.35 (0.19-0.55), specificity = 0.83 (0.78-0.88), and PPV = 0.20. The combination of low cut-off urethritis and microscopic cervicitis was present in 57 cases (19%) with a sensitivity of 0.48 (0.30-0.67), specificity of 0.84 (0.79-0.88), PPV 0.26 (0.16-0.40), NPV 0.93 (0.89-0.96) NND 3, and 15 true positive for Ct (Table 3).

For predicting a positive Mg or Uu, low cut-off urethritis had a sensitivity of 0.46 (0.19-0.75) and 0.45 (0.33-0.57) and PPV= 0.05 and 0.22, respectively. For Uu, purulent wet mount had the highest sensitivity of 0.60 (0.47-0.72), (data not shown).

## 4. Discussion

PMNLs play a critical role in the host defense through phagocytosis of microbes and release of antimicrobial compounds [11]. In men, the penile urethra is the primary site of infection and confirming symptomatic male urethritis with microscopic detection of PMNL is recommended [8]. In women, microscopy does not have a role in diagnosing the equivalent condition and there is no established microscopic definition of urethritis in women [3], except in some Scandinavian clinics where microscopy of urethral smears from women is routine. Studies from these clinics have consistently shown association between microscopically confirmed urethritis and Ct infection [4, 6, 12, 13].

A study from Gothenburg conducted on 99 females attending due to partner notification of* C. trachomatis* found 53 tested positive for Ct and showed that mucopurulent cervical discharge, cervical bleeding, and finding WBCs> epithelial cells in the vaginal wet mount were all significantly associated with a positive Ct result, whereas an increased number of PMNLs in the stained smears from cervix and urethra were not [14]. However, in the Gothenburg study, plastic loops were used for urethral smear sampling, instead of a metal spatula as used by Falk et al. and by Moi et al. [4, 12, 13], which may explain the difference.

The definition of purulent vaginal wet mount is not standardized. A count of WBCs per microscopic field in a stained vaginal smear or vaginal wet mount may be used. However, the thickness and concentration of a vaginal stained smear or wet mount is difficult to standardize. In our study we defined purulent wet mount as more WBCs than epithelial cells in phase-contrast microscopy of a saline vaginal wet mount [14].

The number of WBCs in vaginal tissue has been observed to be stable throughout the menstrual cycle, as were T cells and macrophages [15]. The presence of neutrophil peptides 1–3 (defensins) is closely correlated with the presence of vaginal WBCs [16]. A recent study demonstrated that proinflammatory cytokines EMMPRIN, MMP-8, and NGAL levels were significantly associated with PMNLs count in vaginal Gram stain samples where the vaginal inflammation was defined as >4 PMNLs /HPF [17]. The number of PMNLs in cervix has been observed to be stable throughout the menstrual cycle [1, 18], but sperms influence the cervical inflammatory response and PMNLs increase sharply in cervix after insemination, caused by increased local production of IL-8 in response to seminal plasma [19, 20]. We observed that female patients with regular partners had significant increased PR ratio of microscopic cervicitis (p=0.001) (Table 1), which was confirmed in the multivariate analyses adjusted by significant pathogens, conditions, and behavioural characteristics (p=0.001) (Table 2). Patients without regular partners were twice as likely to use condoms during sex, compared with those who had regular partners (data not shown). Condoms appear to protect against inflammation, perhaps because of the sperm barrier function. However, self-reporting of condom use may be prone to recall bias.

The cervical Gram stain smear has not been standardized and is regarded by the CDC as unhelpful in diagnosis of cervicitis [3]. However, in a study of 558 women in Sydney, the case definition of cervicitis with microscopy (>30 PMNLs) plus cervical discharge had the highest prevalence ratio, positive predictive value, and specificities but lowest sensitivities for the significant pathogens Ct, Ng, Mg, and Tv [21].

The role of Mg in causing LGTI has been contradictory, but a recent meta-analysis reported that women with Mg detected in the cervix uteri had a significantly increased risk of cervicitis [22]. Furthermore, in a study by Moi et al., Mg detection in first void urine showed a clearer association with high-cut urethritis than Mg detection in cervix [13]. In the present study, we detected a low prevalence of Mg and no significant association between Mg infection and clinical/microscopic criteria for LGTI.

The role of other infectious agents in causing LGTI is less clear. In our study Up and Mh were not correlated with microscopic urethritis, purulent wet mount, or cervicitis. Up was isolated in 80 percent of our patients, without any association to signs of LGTI. Liu et al. found that high bacterial loads of Uu and Up may be associated with cervicitis in women [23].

Bacteria associated with BV have been identified among women with cervicitis. Colonization of the cervix with vaginal microbiotas, Mageeibacillus indolicus, may contribute to the clinical manifestations of cervicitis, whereas the presence of Lactobacillus jenseni is inversely associated [24].

LGTI symptoms, dysuria and vaginal discharge, correspond to microscopic signs of LGTI in our study as well as a NAAT positive Ct but not NAAT positive Mg, Uu, Up, or Mh. Screening asymptomatic patients by multiplex NAAT and testing for other bacteria than Ng and Ct can lead to overtreatment and to promote the development of antibiotic-resistant bacteria [25]. NAAT is helpful in the diagnostic of LGTI, but NAAT should not replace clinical examination and microscopy. Quantitative polymerase chain reaction assay and tests for proinflammatory cytokines in mucous membranes are not routine in clinical practice, so microscopy of PMNLs in the genital mucosa could be useful.

## 5. Conclusion

Single criteria for LGTI, except high cut-off urethritis, had low PPV and should not be used alone as single indicators for syndromic treatment. Clinical cervicitis, which is commonly used as criterion for syndromic treatment, was present in 71 cases, of whom 13 had Chlamydia. The combination of high cut-off urethritis and microscopic cervicitis had a high specificity and PP and was present in 30 cases, with a high prevalence (37%) of Ct.

Microscopic criteria for the diagnosis of LGTI give better indication for presumptive antibiotic treatment than anamnestic and clinical diagnosis alone. However, testing and treatment of positive NAATs for Ng and Ct come in addition to syndromic treatment of LGTI. Microscopic examination requires training, experience, and time but is a less subjective diagnostic procedure than cervicitis diagnosed based on clinical examination alone.

Our study has limitations. The study population may have been biased because the women were not systematically included. The 95% CI intervals were wide, and the study should be repeated with a larger patient number. The vaginal wet-mount microscopy was performed bedside by the physician in charge and may have been influenced by the knowledge of the anamnesis and clinical signs.

## Figures and Tables

**Table 1:** Unadjusted prevalence ratios (PRs) of microscopic inflammatory signs by sociodemographic, biological and behavioral characteristics, and clinical conditions.

Characteristics	n	Total n	Prev %	PMNLs>5 (Urethral smear) PR (95% CI)	p	PMNLs>10 (Urethral smear) PR (95% CI)	p	PMNLs > epit. (Vag. wet mount) PR (95% CI)	p	PMNLs>30 (cervical smear) PR (95% CI)	p
No regular partner	236	375	63%	0.89 (0.72-1.11)	0.302	0.86 (0.56-1.33)	0.502	0.93 (0.63-1.36)	0.695	**0.63 (0.47-0.84) **	**0.002**
Regular or cohabitant partner last 6	139	375	37%	1.11 (0.89-1.38)	0.336	1.16 (0.75 - 1.79)	0.502	1.09 (0.74-1.59)	0.667	**1.59 (1.19-2.11)**	**0.001**
Partner>1 last 6 months	277	367	75%	1.26 (0.94-1.67)	0.109	0.96 (0.58 - 1.57)	0.858	1.08 (0.69-1.70)	0.733	1.23 (0.85-1.78)	0.265
Condom use vaginal sex	122	358	34%	0.82 (0.64-1.04)	0.115	0.77 (0.47 - 1.24)	0.280	**0.58 (0.36-0.92)**	**0.021**	0.78 (0.56-1.10)	0.170
Condom use last time	45	363	12%	1.06 (0.77-1.46)	0.701	0.99 (0.51 - 1.94)	0.981	0.95 (0.53-1.70)	0.867	1.01 (0.65-1.58)	0.932
Symptoms of vaginal discharge	190	375	50%	0.81 (0.65-1.00)	0.059	0.79 (0.52 - 1.22)	0.293	**1.56 (1.06-2.29)**	**0.024**	1.07 (0.80-1.43)	0.630
Dysuria	75	375	20%	**1.29 (1.02-1.63)**	**0.030**	**2.00 (1.30 - 3.08)**	**0.002**	0.98 (0.62-1.57)	0.944	1.13 (0.80-1.60)	0.465
Warts	38	375	10%	1.07 (0.76-1.50)	0.681	**0.13 (0.02 - 0.91)**	**0.040**	0.55 (0.24-1.26)	0.158	1.24 (0.81-1.89)	0.313
Cervical motion tenderness	11	375	3%	**1.99 (1.60-2.47)**	**<0.001**	2.04 (0.90 - 4.59)	0.086	**3.74 (2.59-5.4)**	**<0.001**	1.71 (0.97-2.99)	0.060
Mucopurulent endocervical exudate	36	375	10%	**1.34 (1.01-1.78)**	**0.041**	**1.98 (1.18 - 3.33)**	**0.010**	**3.34 (2.38-4.69)**	**<0.001**	**2.42 (1.84-3.19)**	**<0.001**
Endocervical bleeding easily induced	73	375	19%	1.25 (0.99-1.59)	0.060	1.15 (0.69 - 1.92)	0.594	**2.46 (1.72-3.51)**	**<0.001**	**1.53 (1.12-2.08)**	**0.006**
Cervical edema	22	375	6%	1.17 (0.78-1.74)	0.428	1.25 (0.56 - 2.8)	0.581	**3.67 (2.63-5.11)**	**<0.001**	**2.08 (1.46-2.95)**	**<0.001**
Without clinical finding for cervicitis	121	375	32%	0.80 (0.63-1.03)	0.096	0.86 (0.53 - 1.38)	0.522	**0.32 (0.18-0.58)**	**<0.001**	0.81 (0.58-1.12)	0.215
Chlamydia trachomatis^*∗*^	39	375	10%	**1.84 (1.50-2.25)**	**<0.001**	**3.27 (2.17 - 4.94)**	**<0.0001**	**1.82 (1.16-2.85)**	**0.009**	**2.00 (1.46-2.72)**	**<0.001**
Mycoplasma genitalium^*∗*^	16	372	4%	1.07 (0.65-1.78)	0.767	1.39 (0.58 - 3.34)	0.461	1.37 (0.65-2.91)	0.411	1.57 (0.94-2.63)	0.082
Mycoplasma hominis^*∗*^	90	375	24%	1.02 (0.79-1.31)	0.853	1.29 (0.81 - 2.05)	0.277	1.04 (0.67-1.60)	0.872	0.90 (0.63-1.28)	0.561
Ureaplasma urealyticum^*∗*^	91	375	24%	1.04 (0.82-1.33)	0.753	1.19 (0.74 - 1.90)	0.479	1.37 (0.92-2.04)	0.118	**1.35 (1.00-1.84)**	**0.049**
Ureaplasma parvum^*∗*^	299	375	80%	1.34 (0.97-1.84)	0.066	1.50 (0.81 - 2.79)	0.201	1.31 (0.78-2.19)	0.303	0.85 (0.60-1.19)	0.358
Vaginal candidiasis^*∗∗*^	69	371	19%	1.10 (0.84-1.43)	0.468	**1.66 (1.05 - 2.63)**	**0.030**	**1.95 (1.34-2.85)**	**0.001**	1.21 (0.85-1.71)	0.280
pH>4.5	148	370	40%	1.00 (0.81-1.25)	0.932	0.96 (0.62 - 1.49)	0.870	1.39 (0.96-2.01)	0.085	1.25 (0.93-1.67)	0.133
Whiff pos Amine test	105	365	29%	1.03 (0.81-1.31)	0.773	1.18 (0.75 - 1.87)	0.466	1.37 (0.93-2.01)	0.113	1.04 (0.75-1.44)	0.793
Double infection CT/MG/UU^*∗*^	20	375	5%	**1.42 (1.01-.00)**	**0.046**	**2.33 (1.3 - 4.17)**	**0.005**	**1.82 (1.03-3.22)**	**0.041**	**1.75 (1.14-2.69)**	**<0.001**
Previous STD	174	336	52%	0.83 (0.66-1.04)	0.110	1.16 (0.74 - 1.82)	0.507	0.99 (0.67-1.47)	0.947	1.16 (0.85-1.60)	**0.336**

PMNL: polymorphonuclear leukocyte, ^*∗*^Positive NAAT, and ^*∗∗*^pseudohyphae in vaginal KOH wet mount.

Significant values in bold.

**Table 2:** Adjusted prevalence ratios of microscopic signs of inflammation in urethra, vagina, and cervix cervicitis by significant pathogens, conditions, and behavioral characteristics in the univariate analyses.

Characteristics	PMNLs>5 (Urethral smear) APR (95% CI)	p	PMNLs>10 (Urethral smear) APR (95% CI)	p	PMNLs > epit. (Vag. wet smear) APR (95% CI)	p	PMNLs>30 (cervical smear) APR (95% CI)	p
Chlamydia trachomatis	**1.68 (1.34-2.11)**	**<0.001**	**3.00 (2.08-4.35)**	**<0.001**	**1.91 (1.32-2.67)**	**<0.001**	**1.90 (1.34-2.69)**	**<0.001**
Ureaplasma urealyticum	-	-	-	-	-	-	1.37 (0.97-1.94)	0.078
Double infection CT/MG/UU	1.09 (0.89-1.32)	0.413	*∗*		*∗*		1.05 (0.67-1.65)	0.829
Vaginal candidiasis	-	-	**1.52 (1.11-2.09)**	**0.009**	**2.03 (1.46-2.82)**	**<0,001**	-	-
Warts	-	-	1.00 (0.14-6.97)	1.000	-	-	-	-
Regular partner last 6	1.17 (0.97-1.42)	0.100	-	-	-	-	**1.58 (1.22-2.03)**	**<0.001**
Condom use vaginal sex	-		-	-	1.00 (0.66-1.529	1.000		
Symptoms of vaginal discharge	-	-	-	-	**1.64 (1.15-2.33)**	**0.006**	-	-
Dysuria	1.17 (0.96-1.42)	0.112	**1.66 (1.17-2.36)**	**0.004**	-	-	-	-

APR: Adjusted prevalence ratios.

^*∗*^Double infection was excluded as the data were too sparse.

Significant values in bold.

**Table 3:** Clinical and microscopic criteria for LGTI in the subgroup for Chlamydia trachomatis.

**Single tests and test combination**				**N**	**Sensitivity**	**95% CI**	**Specificity**	**95% CI**	**PPV**	**95% CI**	**NPV**	**95% CI**	**NND**	**True pos.**
Urethra: PMNL≥5				135	0,77	0,59 – 0,90	0,58	0,51 – 0,64	0,18	0,12 – 0,25	0,96	0,91 – 0,98	3	24
Urethra: PMNL≥10				50	0,42	0,25 – 0,61	0,86	0,81 – 0,90	0,26	0,15 – 0,40	0,93	0,89 – 0,96	4	13
Wet smear: WBC>Epith				55	0,35	0,19 – 0,55	0,83	0,78 – 0,88	0,20	0,10 – 0,33	0,92	0,87 – 0,95	5	11
Cervix: PMNL>30				91	0,52	0,33 – 0,70	0,71	0,65 – 0,77	0,18	0,10 – 0,27	0,93	0,88 – 0,96	4	16
Clinical cervicitis				71	0,42	0,25 – 0,61	0,78	0,72 – 0,83	0,18	0,10 – 0,29	0,92	0,87 – 0,95	5	13
Urethra: PMNL≥5	Cervix: PMNL>30			57	0,48	0,30 – 0,67	0,84	0,79 – 0,88	0,26	0,16 – 0,40	0,93	0,89 – 0,96	3	15
Urethra: PMNL≥5	Wet smear: WBC>Epith			34	0,29	0,14 – 0,48	0,90	0,86 – 0,94	0,26	0,13 – 0,44	0,92	0,87 – 0,95	5	9
Urethra: PMNL≥5	Clinical cervicitis			41	0,32	0,17 – 0,51	0,88	0,84 – 0,92	0,24	0,12 – 0,40	0,92	0,88 – 0,95	5	10
Urethra: PMNL≥10	Cervix: PMNL>30			30	0,35	0,19 – 0,55	0,93	0,89 – 0,96	0,37	0,20 – 0,56	0,92	0,88 – 0,95	4	11
Urethra: PMNL≥10	Wet smear: WBC>Epith			17	0,16	0,05 – 0,34	0,95	0,92 – 0,98	0,29	0,10 – 0,56	0,91	0,87 – 0,94	9	5
Urethra: PMNL≥10	Clinical cervicitis			15	0,16	0,05 – 0,34	0,96	0,93 – 0,98	0,33	0,12 – 0,62	0,91	0,87 – 0,94	8	5
Cervix: PMNL>30	Clinical cervicitis			34	0,26	0,12 – 0,45	0,90	0,86 – 0,93	0,24	0,11 – 0,41	0,91	0,87 – 0,94	6	8
Wet smear: WBC>Epith	Clinical cervicitis			30	0,23	0,10 – 0,41	0,91	0,87 – 0,94	0,23	0,10 – 0,42	0,91	0,87 – 0,94	7	7
Wet smear: WBC>Epith	Cervix: PMNL>30			25	0,26	0,12 – 0,45	0,94	0,90 – 0,96	0,32	0,15 – 0,54	0,91	0,87 – 0,94	5	8
Urethra: PMNL≥5	Cervix: PMNL>30	Wet smear: WBC>Epith		20	0,23	0,10 – 0,41	0,95	0,92 – 0,97	0,35	0,15 – 0,59	0,91	0,87 – 0,94	5	7
Urethra: PMNL≥5	Cervix: PMNL>30	Clinical cervicitis		25	0,23	0,10 - 0,41	0,93	0,89 – 0,96	0,28	0,12 – 0,49	0,91	0,87 – 0,94	6	7
Urethra: PMNL≥5	Wet smear: WBC>Epith	Clinical cervicitis		21	0,19	0,07 – 0,37	0,94	0,91 – 0,97	0,29	0,11 – 0,52	0,91	0,87 – 0,94	7	6
Urethra: PMNL≥10	Cervix: PMNL>30	Wet smear: WBC>Epith		12	0,16	0,05 – 0,34	0,97	0,95 – 0,99	0,42	0,15 – 0,72	0,91	0,87 – 0,94	7	5
Urethra: PMNL≥10	Cervix: PMNL>30	Clinical cervicitis		11	0,16	0,05 – 0,34	0,98	0,95 – 0,99	0,45	0,17 – 0,77	0,91	0,87 – 0,94	7	5
Urethra: PMNL≥10	Wet smear: WBC>Epith	Clinical cervicitis		9	0,10	0,02 – 0,26	0,98	0,95 – 0,99	0,33	0,07 – 0,70	0,90	0,86 – 0,93	14	3
Cervix: PMNL>30	Wet smear: WBC>Epith	Clinical cervicitis		18	0,19	0,07 – 0,37	0,95	0,92 – 0,98	0,33	0,13 – 0,59	0,91	0,87 – 0,94	6	6
Urethra: PMNL≥5	Cervix: PMNL>30	Wet smear: WBC>Epith	Clinical cervicitis	15	0,16	0,05 – 0,34	0,96	0,93 – 0,98	0,33	0,12 – 0,62	0,91	0,87 – 0,94	8	5
Urethra: PMNL≥10	Cervix: PMNL>30	Wet smear: WBC>Epith	Clinical cervicitis	7	0,10	0,02 – 0,26	0,98	0,96 – 1,00	0,43	0,10 – 0,82	0,90	0,86 – 0,93	12	3

PMNL: polymorphonuclear leucocytes per field in 1000x stained smear.

PPV: positive predictive value. NPV: negative predictive value. NND: number of patients that must be tested in order to produce one correctly classified positive test.

WBC>Epith: more white blood cells than epithelial cells in vaginal wet smear.

## Data Availability

The data used to support the findings of this study are available from the corresponding author upon request.
